# Women with Obesity Are More Likely to Have Long-Term Indwelling Bladder Catheterization in U.S. Nursing Homes

**DOI:** 10.1093/geroni/igab046.3205

**Published:** 2021-12-17

**Authors:** John Harris, Mary Ackenbom, Alison Trinkoff, Steven Handler, John Engberg, David Wolf, Nicholas Castle

**Affiliations:** 1 University of Pittsburgh, Pittsburgh, Pennsylvania, United States; 2 University of Maryland, Baltimore, Maryland, United States; 3 RAND, Pittsburgh, Pennsylvania, United States; 4 Bellarmine, Louisville, Kentucky, United States; 5 West Virginia University, Allison Park, Pennsylvania, United States

## Abstract

Reducing indwelling catheters and increasing clean intermittent catheterization is a key element of effective infection control and maintaining functional independence in nursing homes. Nursing care is often more difficult as obesity increases, leading to more nursing care or equipment to provide care. We hypothesized that nursing homes are more likely to use indwelling catheters for people with obesity because indwelling catheterization likely eases the nursing burden of toileting and personal hygiene care for residents with obesity. The study design was a retrospective cohort study of U.S. nursing home female residents in Minimum Data Set in 2013. Obesity and normal weight (the reference group) were categorized using National Institutes of Health criteria. Indwelling and intermittent bladder catheterization was defined during periodic assessment of residents. We modeled the outcomes using logistic regression using a robust variance estimator. Model covariates included obesity category, resident age, dementia status, comatose status, Stage 3 or 4 pressure ulcers, and the number of activities of daily living deficits. The study cohort included 1,068,388 female residents in 15,230 nursing homes. Obesity (BMI ≥ 30 kg/m2) prevalence was 31.9%. The prevalence of indwelling catheterization was 5.2% and of intermittent catheterization was 0.4%. The odds ratio of indwelling catheter use for obese residents varied from 1.05 to 1.74 (all with p-values <0.001), whereas the odds ratio. of intermittent catheter use varied from 0.84 to 0.46 (all with p-values <0.01) compared to residents of normal weight. Increasing obesity is independently associated with increased long-term indwelling bladder catheterization and decreased intermittent catheterization.

